# Mr. Ring's Addition to His Paper in Our Last Number

**Published:** 1817-02

**Authors:** 


*For the London Medical and Physical Journal.*

Mr. Ring's *Addition to his Paper in our last Number.*

AFTER seeing notice, in the last Number of your Journal, that the subject of discussion between Dr. Kinglake and me must now close, it is with reluctance I am compelled to solicit the admission of a few lines.

When I read Dr. Kinglake's complaint against me, a variety of unavoidable avocations prevented me from examining my answer to him with due attention. I now observe, that, in a former part of it, I have alluded to a passage from his Inaugural Dissertation, quoted by himself in your Journal; but, having then never entertained a doubt that he was a regular physician, it made the less impression on my mind; and, when I heard the unfounded report to the contrary, I did not recollect this circumstance.

I am happy, however, in reflecting, that it is a mistake which admits of being so easily rectified; and, that, when rectified, it can only prove injurious to myself.

*New-street, Hanover-square;*
*Jan.* 3, 1817.

